# Supplementary material for: Theory of topological superconductivity and antiferromagnetic correlated insulators in twisted bilayer WSe2
Source: Nat Commun. 2025 Oct 28;16:9525. doi: 10.1038/s41467-025-64519-3 (PMC12569151; doi:10.1038/s41467-025-64519-3)
Supplement: Supplementary file 1 — Supplementary Information [file 41467_2025_64519_MOESM1_ESM.pdf]

Supplementary Information for 'Theory of Topological Superconductivity and Antiferromagnetic Correlated Insulators in Twisted Bilayer WSe_2'

Chuyi Tuo,[1,*] Ming-Rui Li,[1,2,*] Zhengzhi Wu,[1,3] Wen Sun,[1] and Hong Yao[1,†]

[1]*Institute for Advanced Study, Tsinghua University, Beijing 100084, China*
[2]*Department of Physics, Princeton University, Princeton, New Jersey 08544, USA*
[3]*Rudolf Peierls Centre for Theoretical Physics, Parks Road, Oxford, OX1 3PU, UK*

(Dated: September 1, 2025)

I. Hopping Parameters of the Tight-Binding Model

In this section, we provide additional information of the tight-binding model hopping parameters we adopted in the main text, derived from direct Wannierization of the continuum model for 3.65° $\mathrm{tWSe_2}$ at $V_z = 0$. Fig. S1 illustrates the symmetry inequivalent representative hopping bonds up to 5th-nearest-neighbor, and we provide the hopping parameters for K valley on these representative bonds as well as the onsite potentials as:

$$\begin{aligned}
&t_0^A = t_0^B = -15.26 \text{ meV}, \quad t_0^C = -42.89 \text{ meV} \\
&t_1^{BC} = 7.12 \text{ meV}, \quad t_{\sqrt{3}}^{CC} = 2.76 \text{ meV}, \quad t_2^{AC} = 1.15 \text{ meV} \\
&t_{\sqrt{7}}^{AC} = 3.01 e^{i0.965\pi} \text{ meV}, \quad t_3^{CC} = 0.43 \text{ meV} \\
&t_1^{AB} = -4.99 \text{ meV}, \quad t_{\sqrt{3}}^{BB} = 5.16 e^{i0.618\pi} \text{ meV} \\
&t_2^{AB} = 0.7 \text{ meV}, \quad t_{\sqrt{7}}^{AB} = 0.78 \text{ meV}, \quad t_3^{BB} = -0.31 \text{ meV}
\end{aligned} \tag{S1}$$

where $t_d^{\alpha\beta}$ represents the hopping parameter from β sublattice to α sublattice with distance d (set NN with $d = 1$), and t_0^{α} denotes the onsite potential of α sublattice.

There are several remarks on the symmetry properties of these hopping parameters. First, hopping parameters related by C_{3z} symmetry will (will not) be identical if α, β have identical (different) C_{3z} eigenvalues (see main text). Second, $C_{2y}\mathcal{T}$ and $\mathcal{I}$ symmetries constrain most but not all hopping parameters to be real. Third, the hopping parameters for K and $-K$ valley are related by time-reversal symmetry $\mathcal{T}$. Last, unlike the single-band moiré Hubbard model, where emergent spin-valley SU(2) symmetry exists at $V_z = 0$, the spin-valley symmetry here remains U(1) due to the presence of complex hopping parameters.

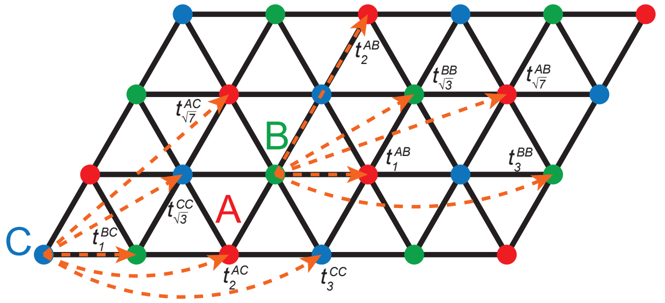

FIG. S1. Illustration of the representative hopping bonds up to 5th-nearest-neighbor.

II. Displacement Field Dependence of the Wannier Functions

The layer-polarization Wannierization scheme can be applied not only to $\mathrm{tWSe_2}$ at zero displacement field, but also at finite displacement fields. Fig. S2 illustrates the density distribution of Wannier functions obtained from the continuum model with the displacement field set to $V_z = 20$ meV (see Eq. (2) in the main text).

Compared with the Wannier functions at zero displacement field (Fig. 1(b) in the main text), we find that the A and B Wannier functions remain almost unchanged (A (B) becomes slightly more localized (extended)) and remain

strongly polarized in opposite layers. In contrast, the C Wannier function exhibits a more noticeable redistribution though remaining layer hybridized. This behavior can be simply understood as follows: the A and B Wannier functions are approximate eigenstates of the displacement field due to their strong layer polarization, while the C Wannier function is not. Nevertheless, we neglect this modification of the C orbital in the main text, as it lies far from the Fermi surface and does not play a significant role in the low-energy physics.

To further justify this approximation, we compute the onsite chemical potential terms t_0^α of the Wannier functions at $V_z = 20$ meV: $t_0^A = -7.77$ meV, $t_0^B = -22.72$ meV, and $t_0^C = -41.10$ meV. These results confirm that the C orbital remains energetically well separated from the low-energy physics. Moreover, we can extract the effective displacement field $\mathcal{V}_z = t_0^A - t_0^B = 14.95$ meV in our tight-binding model (see Eq. (5) in the main text). The fact that $\mathcal{V}_z$ is slightly smaller than the original V_z can be attributed to the A and B Wannier functions not being perfectly layer polarized. These analyses confirm the robustness of the assumptions underlying our model.

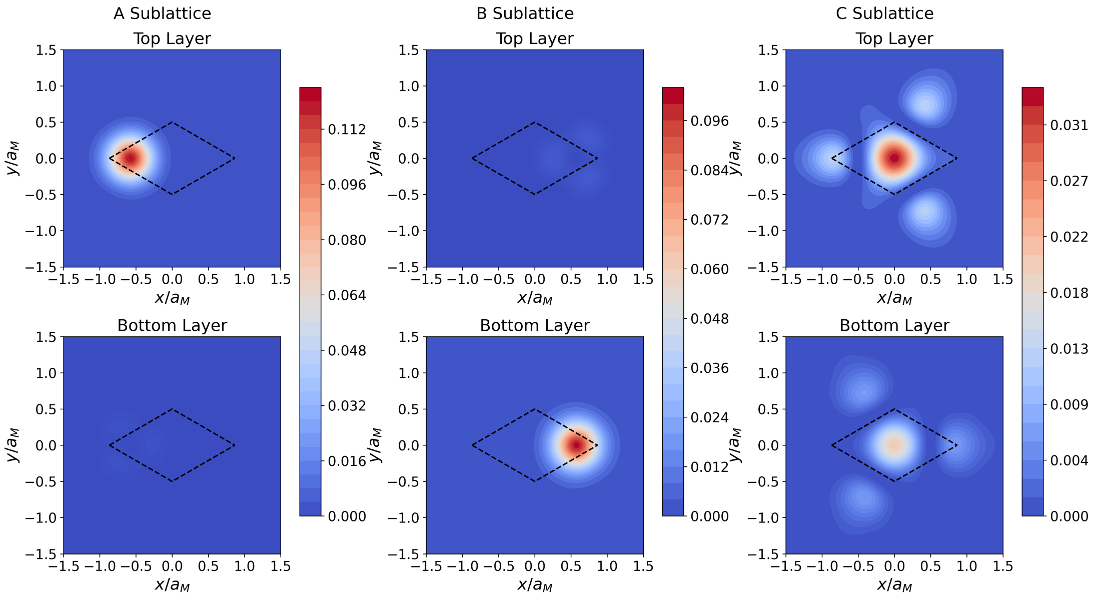

FIG. S2. Density distribution of Wannier functions on two layers at $V_z = 20$ meV, with unit cell shown as dashed line.

III. Mean-field Results with Different Interacting Parameters

In the main text, we adopt the interacting parameters $U_A = U_B = U_C = 37.5$ meV, $V_2 = 10$ meV or 12.5 meV as representative parameters of 3.65° $tWSe_2$. This section further explores our mean-field model over a broad range of interacting parameters, offering valuable physical insights into the $tWSe_2$ system and providing justifications of the parameter choices.

We start with the discussion of onsite Hubbard interaction. We emphasize that, although symmetries only impose $U_A = U_B$ while allowing U_C to differ, the parameter choice can be simplified by setting $U_A = U_B = U_C = U$, as the C orbital is far from the Fermi surface (see Fig. 2 in the main text). Such simplification reduces independent tuning parameters without altering the essential physical properties, making the qualitative physics more transparent. Fig. S3 (a,b) illustrates the AFM energy gain per hole and the mean-field charge gap with $U_A = U_B = 37.5$ meV with varying U_C, showing only minor differences (especially for the small displacement field regime close to the SC phase) even when U_C is tuned over a relatively large range. To gain further insights into the strength of the Hubbard interaction, we then keep $U_A = U_B = U_C = U$ and vary the Hubbard U from 25 meV to 45 meV. The results are shown in Fig. S3 (c,d), where both the AFM energy gain and the charge gap increase with U as expected. Based on these results, we conclude that U in the range of $35 \sim 40$ meV is appropriate, where smaller U weakens the insulating behavior while larger U stabilizes AFM ground state over SC for small displacement field.

We now briefly comment on the values of the NNN attraction V_2. While an infinitesimal attraction V_2 should be sufficient to induce SC tendency (due to the Cooper instability), a relatively large $V_2 = 10$ or 12.5 meV is adopted in the main text. This choice aims to provide a better explanation for the continuous SC-insulator transition in the

experiment. In the main text, we attribute such continuous transition to disorder, which requires our mean-field results (without disorder) either exhibit a weak first-order direct SC-AFM insulator transition ($V_2 = 12.5$ meV), or feature a small intermediate AFM metal phase ($V_2 = 10$ meV). A weaker V_2 results in extended intermediate phase, while a larger V_2 will cause strong direct first-order transition, which is challenging to make continuous even when disorder is considered.

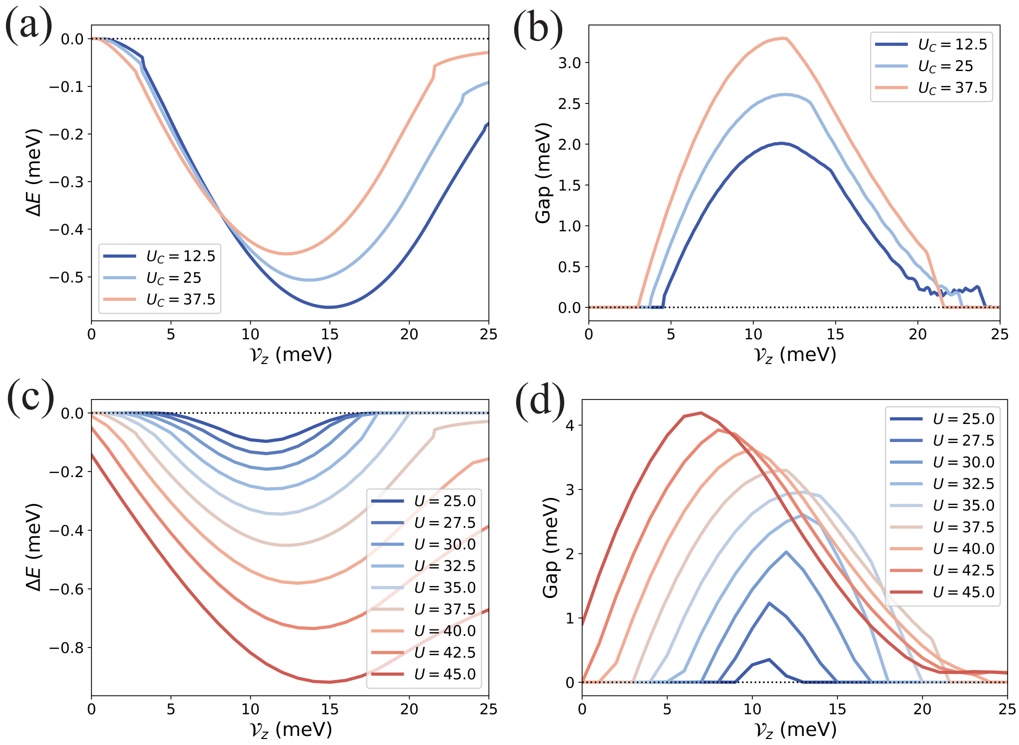

FIG. S3. (a,b) The AFM energy gain per hole and the mean-field charge gap with $U_A = U_B = 37.5$ meV and different U_C. (c,d) The AFM energy gain per hole and the mean-field charge gap with various $U_A = U_B = U_C = U$.

IV. Interacting Parameters Directly from Wannier Functions

In addition to determining the interacting parameters by comparing the mean-field results with the experiment, these parameters can also be directly obtained from the Wannier functions. Given the considerable uncertainties in the microscopic mechanism of attractions, here we primarily focus on the more well-understood dual-gate screened Coulomb repulsion:

$$\begin{aligned} H_{\text{dual-gate}} &= \frac{1}{2A} \sum_{k,k',q,l,l',\tau,\tau'} V_0(q)\, c^\dagger_{k+ql\tau} c^\dagger_{k'-ql'\tau'} c_{k'l'\tau'} c_{kl\tau} \\ V_0(q) &= \pi\xi^2 V_\xi \frac{\tanh(\xi|q|/2)}{\xi|q|/2}, \quad V_\xi = \frac{e^2}{4\pi\epsilon_0\epsilon_r\xi} \end{aligned} \tag{S2}$$

where $c_{kl\tau}$ annihilates a plane wave state with momentum k in layer l and spin/valley τ, A denotes the total area of the system, ξ is the distance between the two metal gates, e is the elementary charge, and ϵ_0 (ϵ_r) correspond to the vacuum (relative) dielectric constant.

Fig. S4 illustrates the interacting parameters obtained by expanding the dual-gate screened Coulomb interaction onto the Wannier functions, using an experimentally relevant gate distance of $\xi = 10$ nm. Here, $V_d^{\bullet\alpha}$ denotes the interacting parameter between a Wannier function on α sublattice and another Wannier function at distance d apart on the same representative bond shown in Fig. S1 (set NN with $d = 1$, and $d = 0$ corresponds to the onsite Hubbard interaction U_α). The dielectric constant ϵ_r is determined by setting $U_{A/B} = 37.5$ meV. We also plot the dual-gate

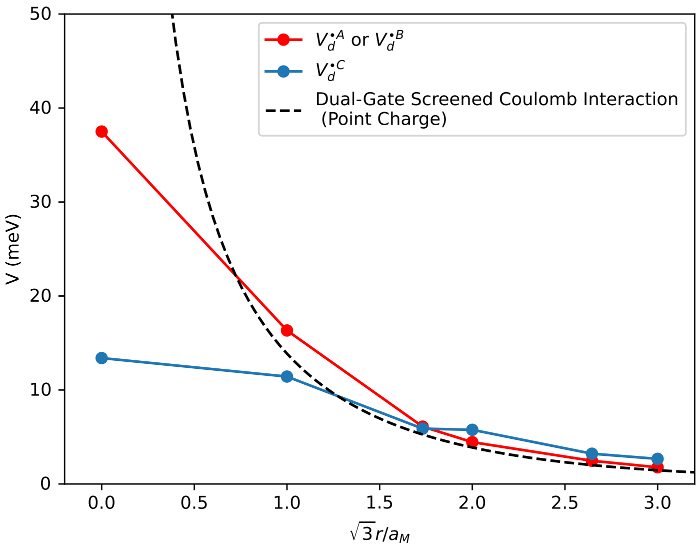

FIG. S4. Interacting parameters obtained by expanding the dual-gate screened Coulomb interaction with $\xi = 10$ nm onto the Wannier functions. The black dashed line represents the dual-gate screened Coulomb interaction for two point charges.

screened Coulomb interaction in real space $V(r)$ (i.e., the Fourier transformation of $V_0(q)$), where the interacting parameters closely follow its trend at large distances. $V(r)$ can be written in summation form as:

$$V(r) = V_\xi \sum_{n=-\infty}^{+\infty} \frac{(-1)^n}{\sqrt{(r/\xi)^2 + n^2}} \tag{S3}$$

which can be physically interpreted as the sum of all contributions from image charges induced by two metal gates.

Here, we offer some additional remarks on these interacting parameters. First, to ensure our mean-field results match the experiment, the dielectric constant is determined to be $\epsilon_r \sim 21.4$ (for $U_{A/B} = 37.5$ meV, as in the main text). The relatively large value of ϵ_r can be attributed to additional screening effects in $tWSe_2$ systems beyond dual-gate screening, including contributions from the dielectric environment or higher-energy bands. Second, we still adopt $U_A = U_B = U_C$ in the main text, though the Hubbard interaction U_C is considerably smaller than $U_{A/B}$ in Fig. S4 (which can be naturally understood by the extension of the Wannier functions, see Fig. 1(b) in the main text). The main reason is that the Wannier functions obtained by the layer-polarization Wannierization scheme are known to be not maximally localized. In particular, the (not so localized) C Wannier function will become more localized if we further optimize its spread functional, resulting in a significantly larger U_C (closer to $U_{A/B}$). Moreover, as already illustrated in Fig. S3(a,b), changing U_C does not affect the main physics of the system, since the C Wannier function is away from the Fermi surface. Third, it is reasonable to retain only the onsite Hubbard interaction while neglecting further neighbor interactions. As shown in Fig. S4, interactions beyond NN are negligibly small, allowing us to safely discard them. Although the NN interaction (from directly expanding the dual-gate Coulomb interaction) has considerable strength (with $V_1^{AB}/U_{A/B} \sim 0.44$), we argue that the effective V_1 should be significantly reduced when accounting for possible attraction mechanisms or additional screening effects beyond dual-gate screening. Moreover, further minimization of the Wannier function spread can further enhance the Hubbard U. Therefore, our model retaining only onsite Hubbard repulsion should provide a good starting point for understanding the physical properties of $tWSe_2$.

V. Attractive Interactions from Random Phase Approximation Analysis

In this section, we provide a microscopic justification of the phenomenological attraction adopted in the main text, using a random phase approximation (RPA) analysis. The RPA method captures collective screening effects by summing the infinite series of bubble diagrams in the particle-hole channel, which can result in effective attractive

interactions in certain real-space regions. In moiré superlattice systems, due to the small size of moiré Brillouin zone, Umklapp scattering must be treated explicitly in the RPA calculation, as we detailed below.

Here, we consider the RPA screening effects of dual-gate screened Coulomb interaction in Eq. (S2) projected to the topmost moiré band. The band projected density operator ρ_{q+g} (where q is in moiré Brillouin zone, g is reciprocal wave vector) is given by:

$$\rho_{q+g} = \sum_{k,\sigma} \Lambda_{g\sigma}^{k+q,k} c_{k+q,\sigma}^{\dagger} c_{k,\sigma} \tag{S4}$$

where $\Lambda_{g\sigma}^{k+q,k}$ is the form factor of the projected density operator, defined by:

$$\Lambda_{g\sigma}^{k_1,k_2} = \sum_{g',l} z_{g+g',l,\sigma}^{*}(k_1) z_{g',l,\sigma}(k_2) \tag{S5}$$

with the definition of $z_{g,l,\sigma}(k) = \langle g, l | u_{k,\sigma}\rangle$, where $|g, l\rangle$ is the plane wave state with momentum g at layer l, and $|u_{k\sigma}\rangle$ is the periodic part of the Bloch state with momentum k and spin/valley σ for the topmost moiré band.

There are several symmetry properties of the form factor $\Lambda_{g\sigma}^{k_1,k_2}$ that can be useful:

$$\Lambda_{g\sigma}^{k_1,k_2} = \Lambda_{g-g'\sigma}^{k_1+g',k_2} = \Lambda_{g+g'\sigma}^{k_1,k_2+g'} \tag{S6}$$

$$\Lambda_{g\downarrow}^{k_1,k_2} = \left[\Lambda_{-g\uparrow}^{-k_1,-k_2}\right]^{*} \tag{S7}$$

$$\Lambda_{g\sigma}^{k_1,k_2} = \left[\Lambda_{-g\sigma}^{k_2,k_1}\right]^{*} \tag{S8}$$

where, Eq. (S6) requires the periodic gauge $z_g(k + g') = z_{g+g'}(k)$, and it is particularly useful for relating arbitrary momentum k_1, k_2 back to the first Brillouin zone. Eq. (S7) requires the time-reversal symmetric gauge $z_{\downarrow}(k) = z_{\uparrow}^{*}(-k)$, which can relate the spin down quantities to the corresponding spin up ones.

Having projected the density operator to the topmost band, the particle-hole susceptibility $\Pi(i\omega_m, q)_{g,g'}$ is defined by the time-ordered density correlation function:

$$\begin{aligned}\Pi(i\omega_m, q)_{g,g'} &= \frac{1}{A}\int_0^{\beta} d\tau e^{i\omega_m\tau} \int dr dr' e^{-i(q+g)r} e^{i(q+g')r'} \Pi(r, r', \tau) \\ &= -\frac{1}{A}\int_0^{\beta} d\tau e^{i\omega_m\tau} \langle T_\tau \rho_{q+g}(\tau)\rho_{-q-g'}(0)\rangle\end{aligned} \tag{S9}$$

Since there are only discrete lattice translation symmetries $\Pi(r + a, r' + a, \tau) = \Pi(r, r', \tau)$ (where a is some moiré lattice vector), momentum is only conserved modulo g. The above equation treats the Umklapp scattering structure explicitly and exactly.

Substitute the projected density operator Eq. (S4) into the above equation, we can evaluate the particle-hole susceptibility exactly:

$$\begin{aligned}\Pi(i\omega_m, q)_{g,g'} &= -\frac{1}{A}\int_0^{\beta} d\tau e^{i\omega_m\tau} \sum_{kk'\sigma\sigma'} \Lambda_{g\sigma}^{k+q,k} \Lambda_{-g'\sigma'}^{k'-q,k'} \langle T_\tau c_{k+q,\sigma}^{\dagger}(\tau) c_{k,\sigma}(\tau) c_{k'-q,\sigma'}^{\dagger}(0) c_{k',\sigma'}(0)\rangle \\ &= \frac{1}{A}\int_0^{\beta} d\tau e^{i\omega_m\tau} \sum_{k\sigma} \Lambda_{g\sigma}^{k+q,k} \Lambda_{-g'\sigma}^{k,k+q} G_\sigma(k,\tau) G_\sigma(k+q,-\tau) \\ &= \frac{1}{A}\sum_{k\sigma} \frac{n_{k\sigma} - n_{k+q\sigma}}{i\omega + \epsilon_{k\sigma} - \epsilon_{k+q\sigma}} \Lambda_{g\sigma}^{k+q,k} \Lambda_{-g'\sigma}^{k,k+q} \\ &= \frac{1}{A}\sum_{k\sigma} \frac{n_{k\sigma} - n_{k+q\sigma}}{i\omega + \epsilon_{k\sigma} - \epsilon_{k+q\sigma}} \Lambda_{g\sigma}^{k+q,k} \left[\Lambda_{g'\sigma}^{k+q,k}\right]^{*}\end{aligned} \tag{S10}$$

where $G_\sigma(k, \tau) = -\langle T_\tau c_\sigma(\tau) c_\sigma^{\dagger}(0)\rangle$ is the single particle green's function, $\epsilon_{k\sigma}$ is the band dispersion and $n_{k\sigma}$ is the Fermi-Dirac distribution.

Defining the bare interaction V_0 as a diagonal matrix of g:

$$V_0(q)_{g,g'} = V_0(q+g)\delta_{g,g'} \tag{S11}$$

Then, the RPA screened interaction follows directly from the standard Dyson series summation (summation over bubble diagrams), where all the quantities should be understood as matrix with index g, g':

$$\begin{aligned} V^{RPA}(i\omega_m, q) &= V_0(q) + V_0(q)\Pi(i\omega_m, q)V_0(q) + V_0(q)\Pi(i\omega_m, q)V_0(q)\Pi(i\omega_m, q)V_0(q) + \cdots \\ &= [1 - V_0(q)\Pi(i\omega_m, q)]^{-1}V_0(q) \end{aligned} \tag{S12}$$

We adopt $V^{RPA}(i\omega_m = 0, q)$ as the RPA effective interaction. We can then Fourier transform to the real space:

$$\begin{aligned} V^{RPA}(r, r') &= \frac{1}{A}\sum_{q,g,g'} V^{RPA}(i\omega_m = 0, q)_{g,g'} e^{i(q+g)r} e^{-i(q+g')r'} \\ &= \frac{1}{A}\sum_{q,g,g'} V^{RPA}(i\omega_m = 0, q)_{g,g'} e^{iq(r-r')} e^{igr} e^{-ig'r'} \end{aligned} \tag{S13}$$

where it should be emphasize that, the momentum space cutoff of the above Fourier transformation should be taken relatively large, since the dual-gate screened interaction $V_0(q)$ in Eq. (S2) has slow $1/q$ decay at large q.

Fig. S5 illustrates the spatial profile of the RPA effective interaction $V^{RPA}(r, r')$ along the NNN or NN directions. Here, $r = 0$ is located at the Wannier center of A sublattice (i.e. XM region), and the dual-gate distance is fixed at experimental relevant value $\xi = 10$ nm. The point $r/a_M = 1$ for the NNN direction (Fig. S5(a)) and the point $\sqrt{3}r/a_M = 1$ for the NN direction (Fig. S5(b)) corresponds to the Wannier center of the NNN or NN site, respectively. These results allow us to evaluate how strongly the Coulomb repulsion is screened and where the effective attraction occurs.

First, we observe that the RPA mechanism further screens the dual-gate screened Coulomb interaction, leading to even more enhanced localization of the repulsive part. This provides further justification of retaining the onsite Hubbard repulsion H_U only in our tight-binding model, as the longer-range repulsions are significantly suppressed.

Moreover, the RPA results reveal that attractive interactions emerge predominantly in the relatively local regions (though some longer-range attractions beyond this range may also occur, but their magnitudes are strongly screened compared with the local ones). Specifically, in Fig. S5(b), a strong attraction is clearly seen near the NN region $\sqrt{3}r/a_M \sim 1$. While in Fig. S5(a) the attraction appears in the range $0.4 \lesssim r/a_M \lesssim 1$ (at $r/a_M = 1$, only weak attraction occurs for relatively large ϵ_r), the NNN interaction can still be attractive (though weaker than the NN attraction) considering the finite spatial extent of the Wannier function. These findings justify the use of relatively local attractive terms in our tight-binding model. While both NN and NNN attraction are possible, our main text mainly focus on the NNN attraction since, as shown below (Fig. S6), it induces much stronger superconductivity, which can be theoretically understood by the sublattice polarization property of the Fermi surface.

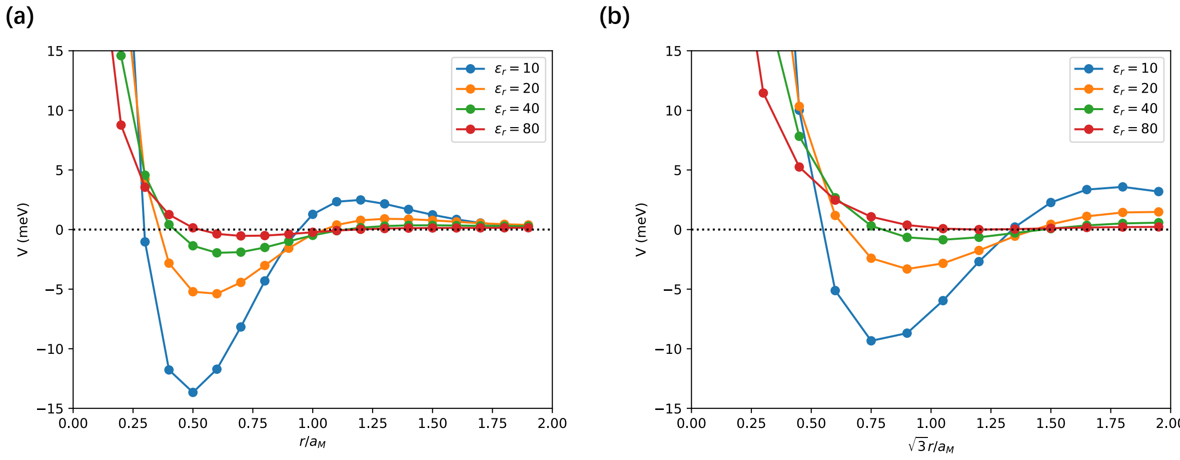

FIG. S5. The RPA effective interactions $V^{RPA}(r, r')$ with r fixed at A sublattice and r' along (a) NNN A-A (b) NN A-B direction with dual-gate distance $\xi = 10$ nm and different dielectric constants ϵ_r.

VI. Derivations and Supplemental Results of Superconducting Mean-Field Analysis

As discussed in the main text, we focus on the interacting tight-binding Hamiltonian of $tWSe_2$ with non-interacting term H_0, displacement field term H_D, onsite Hubbard repulsion H_U, and NNN attraction within A and B sublattices H_{V_2}:

$$H = H_0 + H_D + H_U + H_{V_2} = \sum_{ij\alpha\beta\sigma} (t_{i\alpha j\beta\sigma} - \mu_\alpha \delta_{ij}\delta_{\alpha\beta})\, c^\dagger_{i\alpha\sigma} c_{j\beta\sigma} + \sum_{i\alpha} U_\alpha n_{i\alpha\uparrow} n_{i\alpha\downarrow} - V_2 \sum_{i\sigma\sigma'\alpha} \sum_{\in\{A,B\}} \sum_{\delta\in NNN} n_{i+\delta\alpha\sigma} n_{i\alpha\sigma'}, \tag{S14}$$

where we have combined the chemical potential and displacement field as $\mu_A = \mu - \mathcal{V}_z/2$, $\mu_B = \mu + \mathcal{V}_z/2$ and $\mu_C = \mu$ for simplicity, and other notations are the same as the main text.

The NNN attraction H_{V_2} generally leads to a SC phase, while the onsite Hubbard repulsion H_U typically favors magnetic ordered phase (120° AFM order in this case). Since the SC phase and magnetic ordered phase typically do not coexist, here we assume vanishing magnetic order in the SC mean-field analysis. Thus, according to Eq. (9) in the main text, the mean-field decoupling of H_U only contributes an additional sublattice potential term $\frac{1}{2}\sum_{i\alpha} U_\alpha n_{i\alpha}$.

We decouple the NNN attraction H_{V_2} in the SC channel as:

$$H_{V_2} \approx -V_2 \sum_{i\sigma\sigma'} \sum_{\alpha\in\{A,B\}} \sum_{\delta\in NNN} \left(\tilde{\Delta}^*_{\alpha\sigma\sigma'}(\delta) c_{i\alpha\sigma'} c_{i+\delta\alpha\sigma} + c^\dagger_{i+\delta\alpha\sigma} c^\dagger_{i\alpha\sigma'} \tilde{\Delta}_{\alpha\sigma\sigma'}(\delta) - \tilde{\Delta}^*_{\alpha\sigma\sigma'}(\delta)\tilde{\Delta}_{\alpha\sigma\sigma'}(\delta) \right), \tag{S15}$$

where we have defined the spatially uniform real space pairing order parameter as $\tilde{\Delta}_{\alpha\sigma\sigma'}(\delta) = \langle c_{i\alpha\sigma'} c_{i+\delta\alpha\sigma}\rangle$.

The dependence of order parameter $\tilde{\Delta}_{\alpha\sigma\sigma'}(\delta)$ on NNN bond direction δ is classified according to the SC pairing symmetries. In the main text, we have classified the $S_z = 0$ inter-valley pairing based on the irreducible representation of the point group C_{3v}. Additionally, here we also consider the case that the SC phase breaks C_3 rotation symmetry of the system (i.e. nematic SC) in E representation of C_{3v}, under the assumption that the pairing still preserves one of the three mirror symmetry of C_{3v}. Tab. S1 summarizes the C_{3v} irreducible representations and the form factors $f(\delta)$ (defined by $\tilde{\Delta}_{\alpha\sigma\sigma'}(\delta) = f(\delta)\tilde{\Delta}_{\alpha\sigma\sigma'}$) for each pairing symmetries we considered. And in the following calculations, we will examine each pairing symmetries separately, comparing their energies to determine the most favorable one.

Pairing Symmetry	Irreducible Representation	$f(\delta_1)$	$f(\delta_2)$	$f(\delta_3)$
Mixed s-f wave	A_1	1	1	1
Mixed $p_x \pm ip_y$ - $d_{xy} \mp id_{x^2-y^2}$ wave	E	1	$e^{\pm i\frac{2\pi}{3}}$	$e^{\mp i\frac{2\pi}{3}}$
Mixed p_x - d_{xy} wave	E	0	$\frac{\sqrt{3}}{2}$	$-\frac{\sqrt{3}}{2}$
Mixed p_y - $d_{x^2-y^2}$ wave	E	1	$-\frac{1}{2}$	$-\frac{1}{2}$

TABLE S1. Details of SC pairing symmetries on NNN bonds

With the above symmetry considerations, we take $\sigma' = \bar{\sigma}$ to be opposite to σ for $S_z = 0$ inter-valley pairing, and then Fourier transform the real space mean-field decoupling Eq. (S15) to momentum space:

$$\begin{aligned} &- V_2 \sum_{i\sigma} \sum_{\alpha\in\{A,B\}} \sum_{\delta\in NNN} \left(\tilde{\Delta}^*_{\alpha\sigma\bar{\sigma}}(\delta) c_{i\alpha\bar{\sigma}} c_{i+\delta\alpha\sigma} + c^\dagger_{i+\delta\alpha\sigma} c^\dagger_{i\alpha\bar{\sigma}} \tilde{\Delta}_{\alpha\sigma\bar{\sigma}}(\delta) - \tilde{\Delta}^*_{\alpha\sigma\bar{\sigma}}(\delta)\tilde{\Delta}_{\alpha\sigma\bar{\sigma}}(\delta) \right) \\ =& \sum_{k\sigma} \sum_{\alpha\in\{A,B\}} \left(\Delta^*_{\alpha\sigma\bar{\sigma}}(k) c_{-k\alpha\bar{\sigma}} c_{k\alpha\sigma} + c^\dagger_{k\alpha\sigma} c^\dagger_{-k\alpha\bar{\sigma}} \Delta_{\alpha\sigma\bar{\sigma}}(k) \right) + NV_2 \sum_{\sigma} \sum_{\alpha\in\{A,B\}} \sum_{\delta\in NNN} \tilde{\Delta}^*_{\alpha\sigma\bar{\sigma}}(\delta)\tilde{\Delta}_{\alpha\sigma\bar{\sigma}}(\delta) \end{aligned} \tag{S16}$$

where we have defined the momentum space gap function $\Delta_{\alpha\sigma\bar{\sigma}}(k)$ as $\Delta_{\alpha\sigma\bar{\sigma}}(k) \equiv -V_2 \tilde{\Delta}_{\alpha\sigma\bar{\sigma}} \sum_{\delta\in NNN} f(\delta) e^{-ik\delta}$, and the self-consistency relation in momentum space is given by:

$$\tilde{\Delta}_{\alpha\sigma\bar{\sigma}}(\delta) = \frac{1}{N} \sum_k \langle c_{-k\alpha\bar{\sigma}} c_{k\alpha\sigma}\rangle e^{ik\delta}. \tag{S17}$$

Together with the Fourier transformation of $H_0 + H_D$, we can then write the full momentum space mean-field

Hamiltonian into the Bogoliubov-de-Gennes(BdG) form:

$$\begin{aligned} H_{\mathrm{MF}} =& \sum_{k\alpha\beta\sigma} \left(\epsilon_{\alpha\beta\sigma}(k) - \mu_\alpha \delta_{\alpha\beta}\right) c^\dagger_{k\alpha\sigma} c_{k\beta\sigma} + \sum_{k\sigma} \sum_{\alpha\in\{A,B\}} \left(\Delta^*_{\alpha\sigma\bar\sigma}(k) c_{-k\alpha\bar\sigma} c_{k\alpha\sigma} + c^\dagger_{k\alpha\sigma} c^\dagger_{-k\alpha\bar\sigma} \Delta_{\alpha\sigma\bar\sigma}(k) \right) \\ &+ NV_2 \sum_\sigma \sum_{\alpha\in\{A,B\}} \sum_{\delta\in NNN} \tilde\Delta^*_{\alpha\sigma\bar\sigma}(\delta) \tilde\Delta_{\alpha\sigma\bar\sigma}(\delta) \\ =& \frac{1}{2} \sum_k \begin{pmatrix} \boldsymbol{c}^\dagger_{k\uparrow} & \boldsymbol{c}^\dagger_{k\downarrow} & \boldsymbol{c}_{-k\uparrow} & \boldsymbol{c}_{-k\downarrow} \end{pmatrix} \begin{pmatrix} \boldsymbol{\epsilon}_\uparrow(k) - \boldsymbol{\mu} & \mathbf{0} & \mathbf{0} & \boldsymbol{\Delta}_{\uparrow\downarrow}(k) \\ \mathbf{0} & \boldsymbol{\epsilon}_\downarrow(k) - \boldsymbol{\mu} & \boldsymbol{\Delta}_{\downarrow\uparrow}(k) & \mathbf{0} \\ \mathbf{0} & \boldsymbol{\Delta}^\dagger_{\downarrow\uparrow}(k) & -\boldsymbol{\epsilon}^T_\uparrow(-k) + \boldsymbol{\mu} & \mathbf{0} \\ \boldsymbol{\Delta}^\dagger_{\uparrow\downarrow}(k) & \mathbf{0} & \mathbf{0} & -\boldsymbol{\epsilon}^T_\downarrow(-k) + \boldsymbol{\mu} \end{pmatrix} \begin{pmatrix} \boldsymbol{c}_{k\uparrow} \\ \boldsymbol{c}_{k\downarrow} \\ \boldsymbol{c}^\dagger_{-k\uparrow} \\ \boldsymbol{c}^\dagger_{-k\downarrow} \end{pmatrix} \\ &+ \frac{1}{2} \sum_{k\alpha\sigma} \left(\epsilon_{\alpha\alpha\sigma}(k) - \mu_\alpha\right) + NV_2 \sum_\sigma \sum_{\alpha\in\{A,B\}} \sum_{\delta\in NNN} \tilde\Delta^*_{\alpha\sigma\bar\sigma}(\delta) \tilde\Delta_{\alpha\sigma\bar\sigma}(\delta), \end{aligned} \tag{S18}$$

where the pairing matrix is defined as:

$$\boldsymbol{\Delta}_{\sigma\bar\sigma}(k) = \begin{pmatrix} \Delta_{A\sigma\bar\sigma}(k) - \Delta_{A\bar\sigma\sigma}(-k) & 0 & 0 \\ 0 & \Delta_{B\sigma\bar\sigma}(k) - \Delta_{B\bar\sigma\sigma}(-k) & 0 \\ 0 & 0 & 0 \end{pmatrix} \tag{S19}$$

With the mean-field Hamiltonian derived above, we can perform the standard mean-field calculations. We first construct the BdG Hamiltonian in Eq. (S18) using some randomly chosen initial values of the order parameters $\tilde\Delta_{\alpha\sigma\bar\sigma}$, and adjust the chemical potential μ to fit the correct average filling $\nu = -1$ by $n_e = \sum_{k\alpha\sigma} \langle c^\dagger_{k\alpha\sigma} c_{k\alpha\sigma} \rangle$, with the many-body ground state of the BdG Hamiltonian is obtained by filling all quasi-particle states with negative energy. And the ground state expectation value in Eq. (S17) is calculated to update the order parameters for the next iteration. The above process is repeated until the self-consistency is achieved.

Although we have argued in the main text that NNN attraction is more likely to induce stronger SC instability, here we also examine an alternative scenario that the NNN attraction H_{V_2} is replaced by a NN attraction H_{V_1}:

$$H' = H_0 + H_D + H_U + H_{V_1} = \sum_{ij\alpha\beta\sigma} \left(t_{i\alpha j\beta\sigma} - \mu_\alpha \delta_{ij} \delta_{\alpha\beta}\right) c^\dagger_{i\alpha\sigma} c_{j\beta\sigma} + \sum_{i\alpha} U_\alpha n_{i\alpha\uparrow} n_{i\alpha\downarrow} - V_1 \sum_{i\sigma\sigma'} \sum_{\delta\in NN} n_{i+\delta A\sigma} n_{iB\sigma'}. \tag{S20}$$

The SC mean-field analysis for H' closely mirrors that of H we have derived above, where the only differences are the substitution of δ from NNN to NN bonds and the modification of sublattice indices. Therefore, we will omit the derivation here for brevity, and only show the corresponding classification of SC pairing symmetries on NN bonds (only considering chiral SC in E representation) in Tab. S2.

Pairing Symmetry	Irreducible Representation	$f(\delta_1)$	$f(\delta_2)$	$f(\delta_3)$
s wave	A_1	1	1	1
Mixed $p_x \pm ip_y$ - $d_{xy} \mp id_{x^2-y^2}$ wave	E	1	$e^{\pm i\frac{2\pi}{3}}$	$e^{\mp i\frac{2\pi}{3}}$

TABLE S2. Details of SC pairing symmetries on NN bonds

Taking $U_A = U_B = U_C = 37.5$ meV, $V_2 = 10$ meV as in the main text for the NNN Hamiltonian H (see Eq. (S14)), and $V_1 = 10$ meV for the NN Hamiltonian H' (see Eq. (S20)), we perform the mean-field calculations for all pairing symmetries classified in Tab. S1 and Tab. S2. The resulting energy gain per hole for NNN and NN Hamiltonians are illustrated in Fig. S6(a) and (b) respectively.

A direct comparison of the energy gains ΔE reveals that NNN pairings are approximately $2 \sim 3$ orders of magnitude stronger than NN pairings for same attraction strength $V_1 = V_2$. We thus conclude that the NNN pairings should be more dominant than the NN pairings, justifying our main focus of the NNN pairings in the main text. This result is actually straightforward to understand. Due to the time-reversal symmetry $\mathcal{T}$ in both H and H', the Cooper pairs will form between time-reversal-related states. As the time-reversal symmetry $\mathcal{T}$ does not alter sublattice index α, and given that the Fermi surface have relatively strong sublattice polarization property (as shown in Fig. 2(a) in the main text), the system is expected to favor intra-sublattice pairings (e.g. NNN pairings) over inter-sublattice pairings (e.g. NN pairings). Considering the layer polarization property of the A and B sublattices (the main components of the Fermi surface), it is equivalent to say that the tWSe_2 system favors intra-layer pairings.

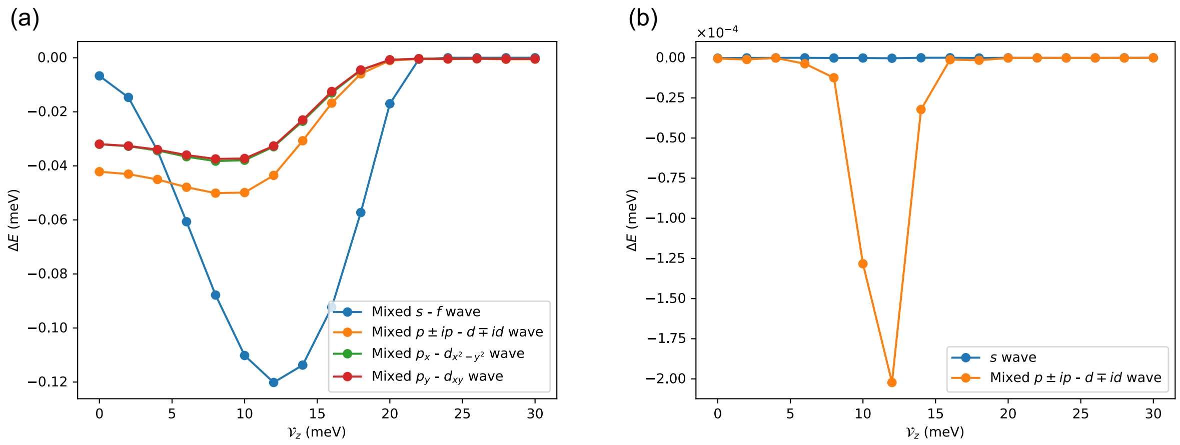

FIG. S6. The energy gain ΔE per hole of the SC phases compared to the symmetric phase with (a) NNN and (b) NN pairings.

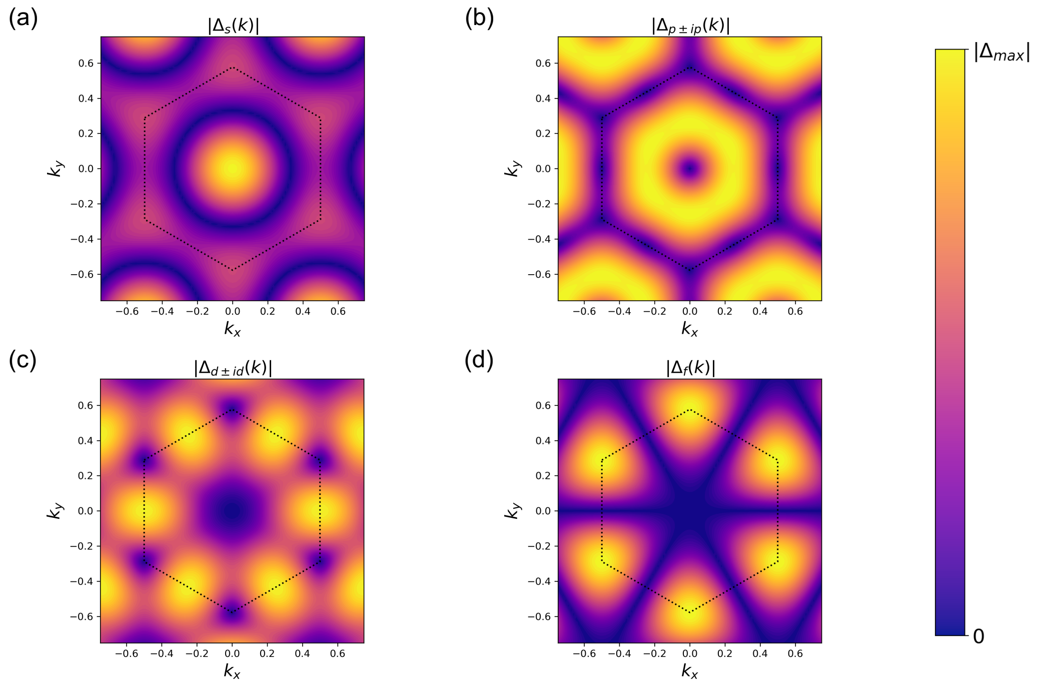

FIG. S7. Momentum space form factor for NNN pairing with (a) s-wave, (b) $p_x \pm ip_y$-wave, (c) $d_{xy} \mp id_{x^2-y^2}$-wave and (d) f-wave symmetry, and the BZ is shown as black dash line in each figure.

Next, we examine the nematic SC orders (with mixed p_x-d_{xy} or p_y-$d_{x^2-y^2}$-wave symmetry) as shown in Fig. S6(a). These nematic SC states have energies considerably higher than the chiral ones under small displacement field. It is expected that some moderate C_3 breaking terms (e.g. strain) may favor such nematic SC over chiral ones. And it should be also noted that these two nematic SC states are nearly (but not exactly) degenerate in energy.

Returning to the non-nematic cases for NNN pairings, one can see that at small $\mathcal{V}_z$ (as discussed in the main text) or large enough $\mathcal{V}_z$ regime (where the energies of different pairing symmetries are close), the chiral mixed $p_x \pm ip_y$ - $d_{xy} \mp id_{x^2-y^2}$-wave symmetry is the most dominant, while at intermediate $\mathcal{V}_z$, the mixed s-f-wave symmetry becomes the strongest one. To understand this, we plot the magnitude of the momentum space form factor for NNN pairings with different partial wave components in Fig. S7. As shown in Fig. S7, the s-wave component has local maximum at γ and $\kappa_\pm$ points of Brillouin zone (BZ) while having ring-shaped minimum in between; the $p_x \pm ip_y$-wave component is large except at γ points and the BZ boundary; the $d_{xy} \pm id_{x^2-y^2}$-wave component has maximum at m points while having minimum at γ and $\kappa_\pm$ points; and the f wave component has maximum at $\kappa_\pm$ points but having minimum

nodal line along γ-m directions.

The energetic of these different SCs can then be understood by combining the Fermi surface structures shown in Fig. 2 in the main text with the form factors shown in Fig. S7. At small displacement field $\mathcal{V}_z$, the Fermi surface is hexagonal shape around the γ point in the BZ, where the chiral $p_x \pm ip_y$ and $d_{xy} \pm id_{x^2-y^2}$ components are the largest and fully gaps the Fermi surface. When increasing $\mathcal{V}_z$, Fig. 2(b) in the main text indicates that B sublattice has dominant DOS, which should be our primary focus. And Fig. 2(a) in the main text clearly illustrates that the B pockets are move towards $\kappa_\pm$ points in the BZ as the displacement field $\mathcal{V}_z$ is increasing, where the s and f-wave components are larger in magnitude and become better in energy. However, as $\mathcal{V}_z$ increases further, the B sublattice Fermi pockets at $\kappa_\pm$ points disappear, leaving the remaining Fermi surface mainly on A sublattice, which is again located at the region where $p_x \pm ip_y$ and $d_{xy} \pm id_{x^2-y^2}$ components are dominant. The above analysis suggests that the combined use of the structure of Fermi surfaces and momentum space pairing form factors provides a clear explanation of the mean-field behaviors of the SC phases.

VII. Derivations of Magnetic Mean-Field Analysis

In this section, we provide a more detailed mean-field analysis for possible magnetic orders of the NNN Hamiltonian H in Eq. (S14). Assuming the absence of SC order (hence H_{V_2} has vanishing mean-field contributions), we mainly focus on the in-plane magnetic channel of H_U as:

$$H_U \approx -\sum_{i\alpha} U_\alpha \left(m_{i\alpha} c^\dagger_{i\alpha\downarrow} c_{i\alpha\uparrow} + m^*_{i\alpha} c^\dagger_{i\alpha\uparrow} c_{i\alpha\downarrow} \right) + \sum_{i\alpha} U_\alpha |m_{i\alpha}|^2 + \frac{1}{2} \sum_{i\alpha\sigma} U_\alpha n_{i\alpha\sigma}, \tag{S21}$$

where $m_{i\alpha} = \langle c^\dagger_{i\alpha\uparrow} c_{i\alpha\downarrow} \rangle = \langle S^x_{i\alpha} \rangle + i \langle S^y_{i\alpha} \rangle$ is the in-plane magnetic order parameter. As discussed in the main text, due to the existence of approximate nesting wave vector $\boldsymbol{Q} = (0, 4\pi/3a_M)$ between spin up and down Fermi surfaces, we first consider the ansatz of 120° AFM order $m_{i\alpha} = m^+_\alpha e^{iQ\cdot R_i} + m^-_\alpha e^{-iQ\cdot R_i} = \sum_\eta m^\eta_\alpha e^{i\eta Q\cdot R_i}$. Performing Fourier transformation, the mean-field Hamiltonian is given by:

$$H_{\mathrm{MF}} = \sum_{k\alpha\beta\sigma} \left(\epsilon_{\alpha\beta\sigma}(k) + \frac{U_\alpha}{2} \delta_{\alpha\beta} - \mu_\alpha \delta_{\alpha\beta} \right) c^\dagger_{k\alpha\sigma} c_{k\beta\sigma} - \sum_{k\alpha\eta} U_\alpha \left(m^\eta_\alpha c^\dagger_{k+\eta Q\alpha\downarrow} c_{k\alpha\uparrow} + m^{\eta *}_\alpha c^\dagger_{k-\eta Q\alpha\uparrow} c_{k\alpha\downarrow} \right) + N \sum_{\alpha\eta} U_\alpha |m^\eta_\alpha|^2, \tag{S22}$$

and the order parameter m^η_α satisfies the self-consistent equation:

$$m^\eta_\alpha = \frac{1}{N} \sum_k \langle c^\dagger_{k\alpha\uparrow} c_{k+\eta Q\alpha\downarrow} \rangle. \tag{S23}$$

Notice that the momentum summation above should be performed in the original BZ with N being the number of original unit cells. And in practice, the folding of BZ, as shown in Fig. 3(d) in the main text, can be easily achieved by a simple relabeling of momentum.

Then, we can perform the standard mean-field calculations as follow. We first construct and diagonalize the mean-field Hamiltonian in Eq. (S22), and filled the lowest energy states until $\nu = -1$. We then use Eq. (S23) to update the order parameters for next iteration. Such process is repeated until self-consistency is achieved, and the numerical results are detailed in the main text.

However, there are also other possible competing orders, one important class being the zero momentum magnetic orders, which do not break the translation symmetry. Since there is no experimental evidence of valley polarization ferromagnetism (i.e. magnetic order in z direction), here we again focus on the in-plane magnetic channel. The decoupling of the Hubbard interaction H_U in momentum space is:

$$H_U \approx -\sum_{k\alpha} U_\alpha \left(m_\alpha c^\dagger_{\alpha k\downarrow} c_{\alpha k\uparrow} + m^*_\alpha c^\dagger_{\alpha k\uparrow} c_{\alpha k\downarrow} \right) + N \sum_\alpha U_\alpha m^2_\alpha + \frac{1}{2} \sum_{k\alpha\sigma} U_\alpha n_{k\alpha\sigma}, \tag{S24}$$

where the in-plane magnetic order parameter is given by $m_\alpha = \frac{1}{N} \sum_k \langle c^\dagger_{k\alpha\uparrow} c_{k\alpha\downarrow} \rangle$. We have numerically checked that such magnetic order is not favored when turning the displacement field on, and we conclude that 120° AFM order is indeed more favorable. Such results can be easily understood since the displacement field splits the Fermi surfaces of spin up and spin down, hence suppress the zero-momentum inter-valley coherent magnetic orders.

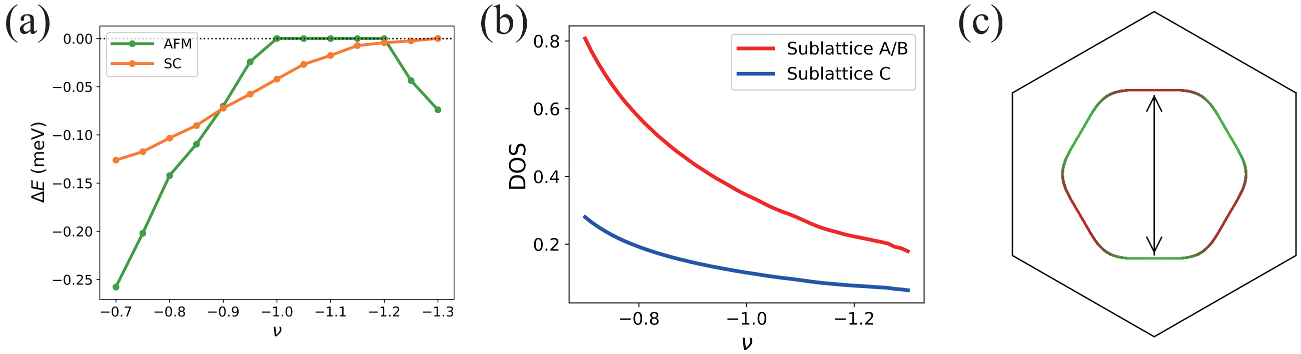

FIG. S8. (a) The energy gain per hole of the AFM order and the mixed $p_x \pm ip_y$ and $d_{xy} \mp id_{x^2-y^2}$-wave SC order. (b) the Fermi surface DOS as a function of filling ν. (c) Fermi surface at $\nu = -1.3$, where the nesting wave vector $\boldsymbol{Q} = (0, \pm 4\pi/3a_M)$ is represented as black arrow.

VIII. Mean-Field Results Away From Filling $\nu = -1$

In the experiment, the 3.65° $tWSe_2$ system exhibits metallic behavior on both sides as the filling deviating from $\nu = -1$. In this section, we present a preliminary theoretical investigation on the doping dependence of $tWSe_2$ near the SC phase through mean-field analysis, demonstrating that our model can also explain the key experimental features when the filling is away from $\nu = -1$. Theoretically, the commensurate $\sqrt{3} \times \sqrt{3}$ AFM order tendency generally persists over a certain range of filling near $\nu = -1$, leading to a metallic phase since the filling factor (in the enlarged unit cell) deviates from the integer value $\tilde{\nu} = -3$. Meanwhile, the SC tendency also remains robust upon doping away from $\nu = -1$, as the time-reversal symmetry remains intact. The actual ground state is determined by the energetics of these two phases.

Fig. S8(a) illustrates our mean-field results for the filling dependence of SC and AFM orders at $\mathcal{V}_z = 0$ near $\nu = -1$, indicating that the SC phase persists only within the range $0.9 \lesssim |\nu| \lesssim 1.2$, while an AFM metal phase emerges beyond this filling range, qualitatively consistent with the experimental observations. To better understand the filling dependence of these orders, we present the Fermi surface DOS as a function of ν in Fig. S8(b), where the DOS is significantly enhanced (suppressed) on the less (more) hole-doped side due to its closer (further) proximity to the van Hove singularity. The Fermi surface DOS provides a natural understanding for the strength of SC order, as well as the enhanced AFM order on the less hole-doped side. In contrast, the emergence of AFM order on the more hole-doped side, as shown in Fig. S8(c), arises from the better nesting condition for the $\sqrt{3} \times \sqrt{3}$ AFM order.

* These two authors contribute equally in this work
† yaohong@tsinghua.edu.cn
